# Supplementary material for: Megakaryocyte- and Platelet-Derived Microparticles as Novel Diagnostic and Prognostic Biomarkers for Immune Thrombocytopenia
Source: J Clin Med. 2022 Nov 16;11(22):6776. doi: 10.3390/jcm11226776 (PMC9698595; doi:10.3390/jcm11226776)
Supplement: Supplementary file 1 [file jcm-11-06776-s001.zip › jcm-1997231-supplementary.pdf]

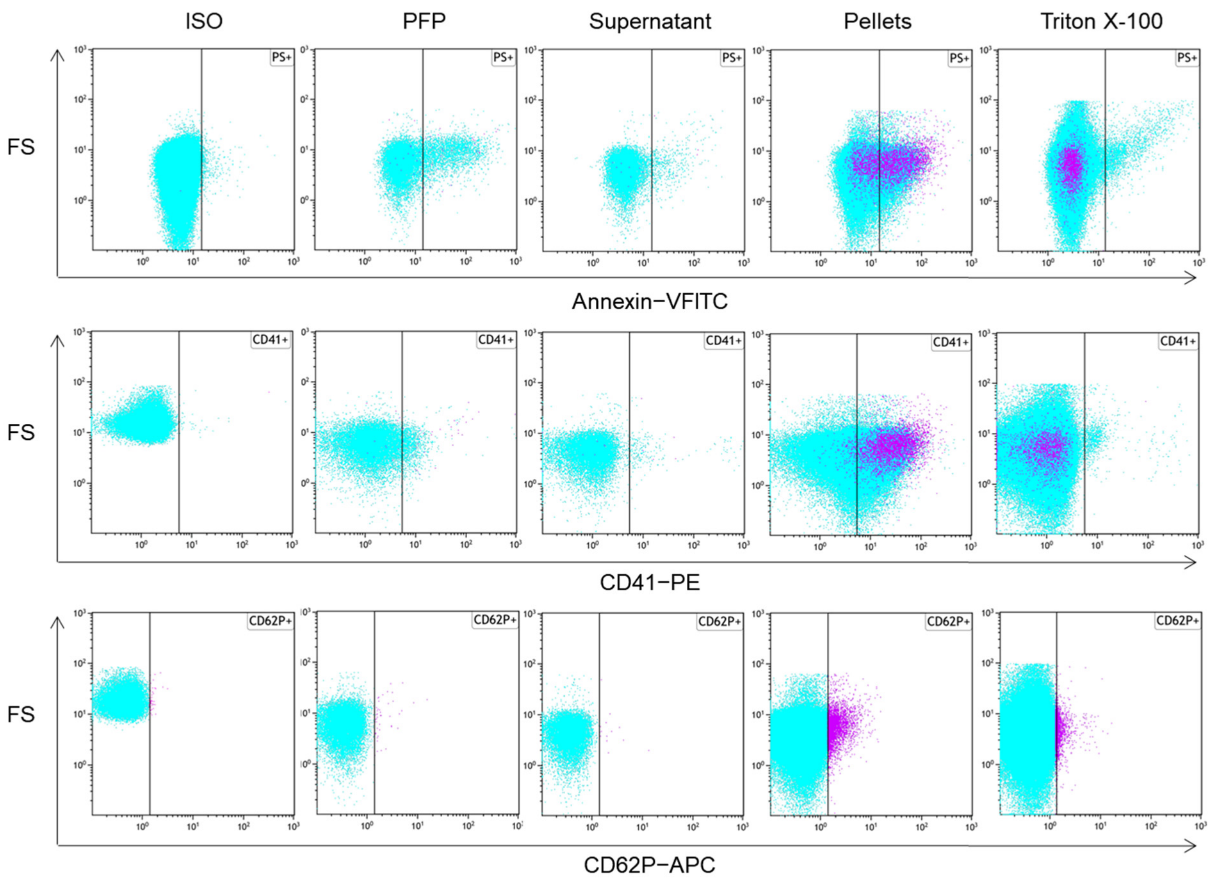

Supplementary Figure S1. Validation of positive MP events by flow cytometry using ultracentrifugation-isolated and Triton X-100-treated MPs. Antibodies specificity was verified by isotype controls. The positive MP events were identified by the differences before and after ultracentrifugation and 2% Triton X-100 treatment. PS, phosphatidylserine. ISO, isotype control. PFP, MPs in processed platelet-free plasma. Supernatant and Pellets, MPs in PFP supernatant and pellets after ultracentrifugation. Triton X-100, MPs in 2% Triton X-100 treated PFP pellets.

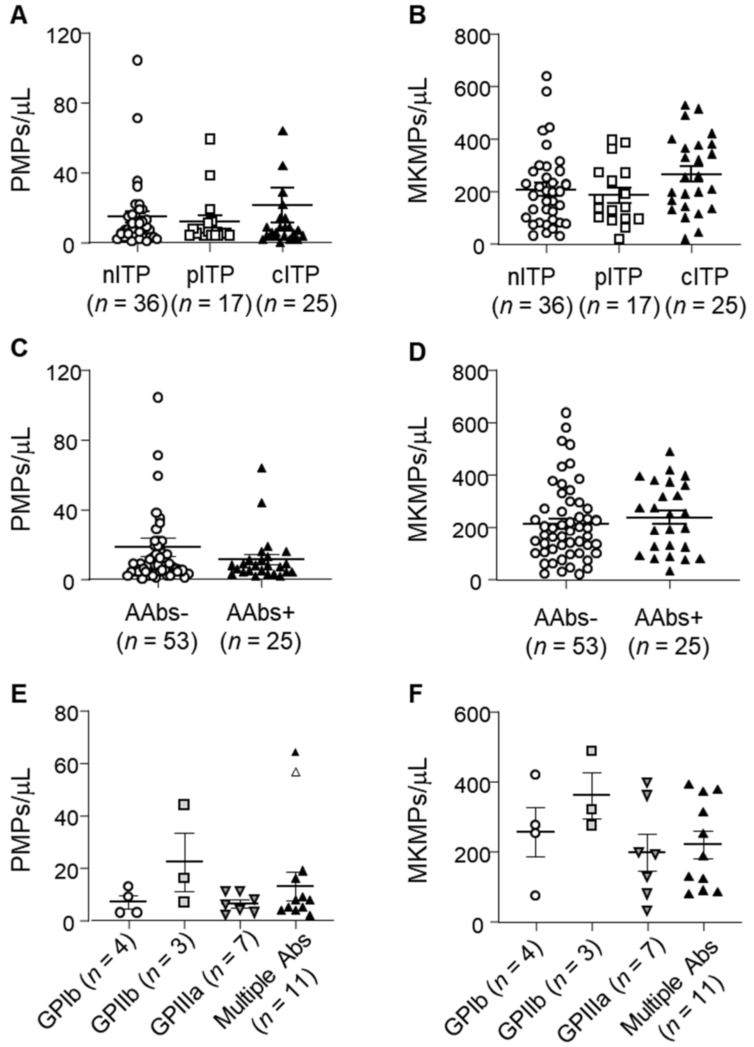

Supplementary Figure S2. The MP levels in ITP subgroups. The PMP and MKMP levels were compared based on the disease courses (**A**, **B**), presence or absence of autoantibodies (**C**, **D**), and antibody subtypes (**E**, **F**). No significant difference was observed among these subgroups.

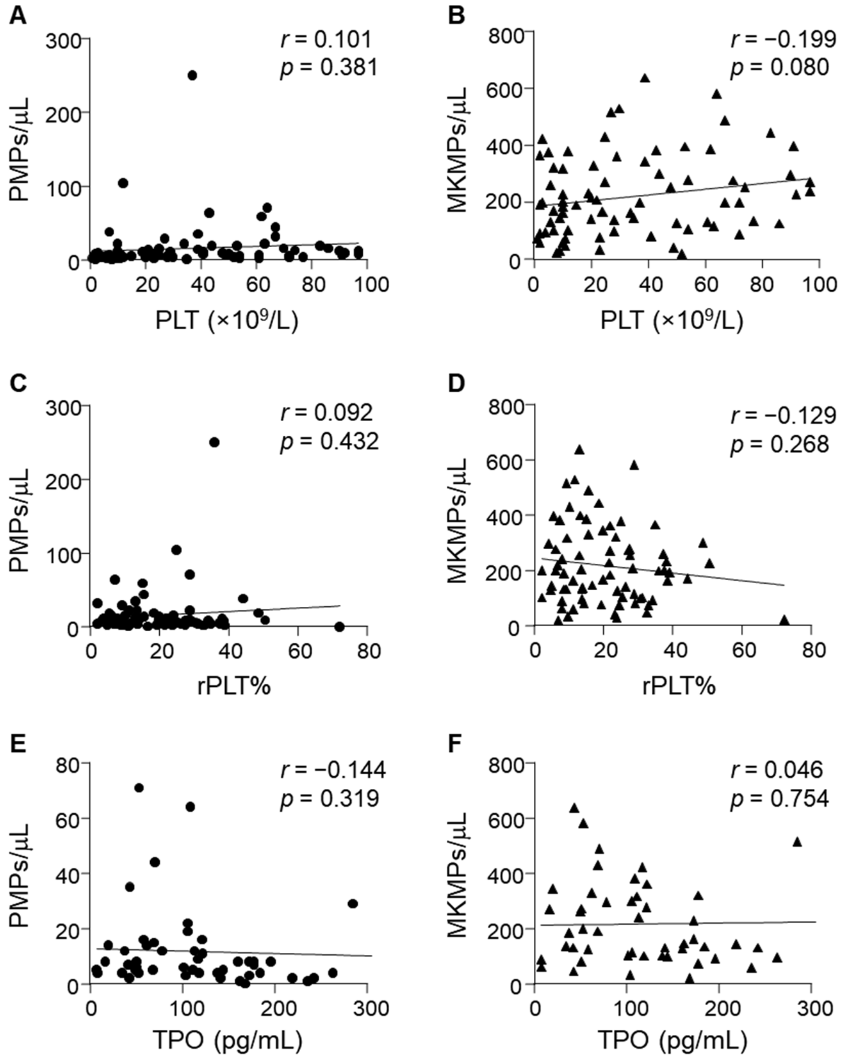

Figure S3. Correlation analysis between MPs levels and ITP-related parameters. The Spearman correlation analysis was performed between plasma MPs levels and platelet counts (PLT) (A, B), percentage of reticulated platelets (rPLT%) (C, D), and TPO levels (E, F) in ITP patients (n = 78). No significant correlation was observed among these parameters.
